# Peripheral predominance of facial idiopathic guttate hypomelanosis in a retrospective observational study: A clinical clue to distinguish from vitiligo

**DOI:** 10.1016/j.jdin.2026.06.020

**Published:** 2026-07-16

**Authors:** Youngbeom Kim, Kihyuk Shin, Hoon-Soo Kim, Hyun-Chang Ko, Byungsoo Kim, Moon-Bum Kim, Jungsoo Lee

**Affiliations:** a Department of Dermatology, School of Medicine, Pusan National University, Busan, Republic of Korea; b Biomedical Research Institute, Pusan National University Hospital, Busan, Republic of Korea; c Department of Dermatology, Pusan National University Yangsan Hospital, Yangsan, Republic of Korea; d Research Institute for Convergence of Biomedical Science and Technology, Pusan National University Yangsan Hospital, Yangsan, Republic of Korea

**Keywords:** distribution, epidemiology, facial, hypopigmentation, idiopathic guttate hypomelanosis, vitiligo

*To the Editor:* Idiopathic guttate hypomelanosis (IGH) is a common acquired leukoderma, more frequently observed in older individuals. It is characterized by multiple, discrete, porcelain-white macules on sun-exposed areas, particularly the forearms and pretibial regions.^1^ Although facial involvement has been reported to be uncommon,^2^ it may pose a diagnostic challenge because of its resemblance to facial vitiligo. However, the distribution pattern of facial IGH has not been well characterized.^3^

We retrospectively reviewed patients diagnosed with facial IGH at 2 tertiary referral centers between August 2010 and July 2022. Diagnosis was made clinically by 2 dermatologists based on characteristic hypopigmented macules that were asymptomatic and stable, with dermoscopic examination revealing white structureless areas with absent pigment network, consistent with IGH. Stability was defined as no change in size, number, or distribution over ≥6 months. In cases of diagnostic discordance, a consensus diagnosis was reached through joint review; cases in which consensus could not be reached were excluded from the study. Vitiligo was excluded based on the absence of complete depigmentation, sharply demarcated borders, and bright chalk-white enhancement on Wood lamp examination. No Koebner phenomenon, confetti-like depigmentation, or leukotrichia were observed. Patients with other causes of facial hypopigmentation were excluded, and only those with high-quality full-face clinical photographs were included. Skin biopsy was not performed given the characteristic clinical and dermoscopic findings. Representative clinical images of facial IGH are shown in Fig 1 and Supplementary Figs 1 to 5, available via Mendeley at https://data.mendeley.com/datasets/4dkjxthn6x/2.

**Fig 1:**
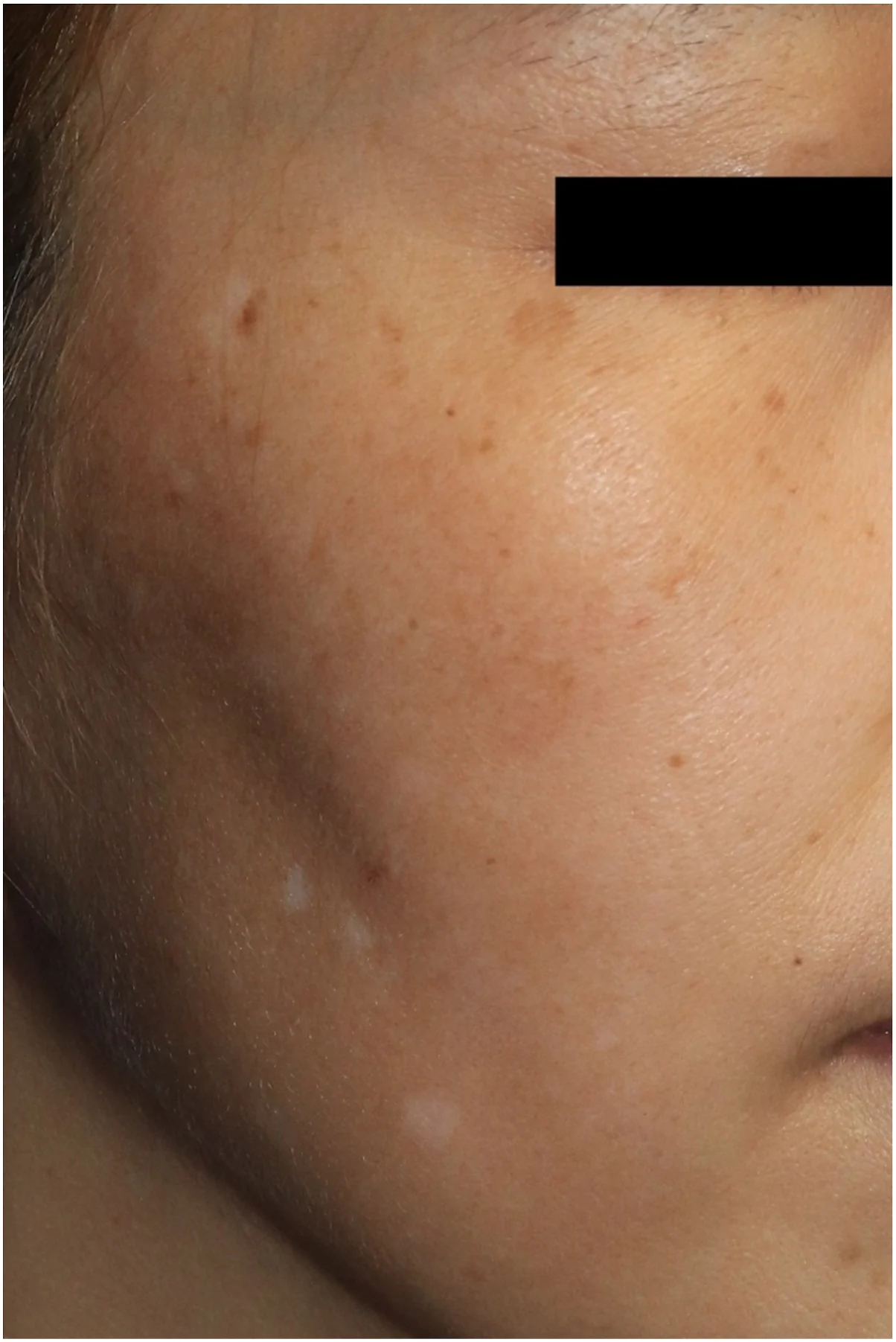
Facial idiopathic guttate hypomelanosis. Multiple small, discrete hypopigmented macules showing peripheral predominance over the lateral cheek, with coexisting solar lentigines.

Each lesion was mapped onto a standardized facial diagram divided into 18 anatomical units. For analysis, these units were grouped into central (periorbital, infraorbital, nasal, and cutaneous lip) and peripheral (forehead, temple, zygomatic, mandibular, and chin) regions. A heatmap was generated to visualize lesion distribution (Fig 2).

**Fig 2:**
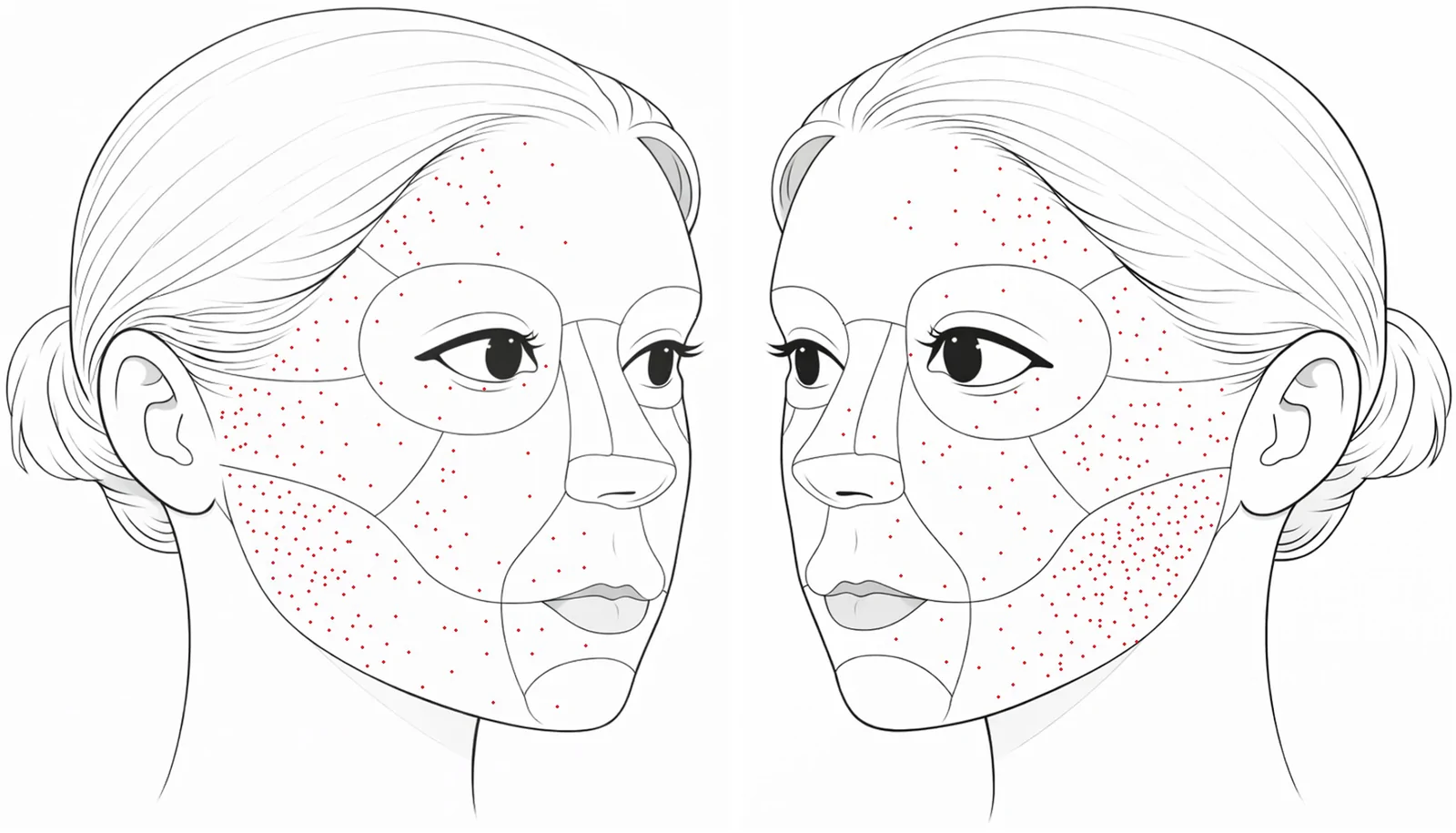
Heatmap showing the distribution of facial idiopathic guttate hypomelanosis, demonstrating a predominant peripheral distribution.

Thirty-three patients were included (mean age, 59.9 ± 8.6 years), with Fitzpatrick skin types III, IV, and V in 16, 15, and 2 patients, respectively. This is consistent with Korean skin phototype distribution. A total of 431 facial IGH lesions were identified (mean, 13.06 lesions per patient). Lesions were most frequently observed in the mandibular region, followed by the zygomatic area. Peripheral regions contained significantly more lesions than central regions (10.45 vs 2.60 lesions per patient, *P* < .05). No significant differences in lesion counts were observed according to Fitzpatrick skin type, Glogau scale, or laterality.

The observed peripheral predominance, which was not associated with the Glogau photoaging scale, suggests that factors other than cumulative ultraviolet exposure may contribute to the distribution of facial IGH.^4^ This distribution contrasts with the typical pattern of facial vitiligo, which commonly involves central and periorificial regions.^5^ Recognition of this distributional pattern may serve as a practical clinical clue to distinguish facial IGH from vitiligo in routine practice.

In conclusion, facial IGH demonstrated a predominant peripheral distribution pattern, particularly involving the mandibular and zygomatic regions. This finding may provide an additional clinical clue for differentiating facial IGH from vitiligo. Given the rarity of facial IGH and the retrospective design of this study, future prospective studies with larger cohorts are warranted to further validate this distributional pattern.

### Declaration of generative AI and AI-assisted technologies in the writing process

The authors confirm that no artificial intelligence (AI) tools, including large language models, were used in the writing, analysis, or preparation of this manuscript.

## Conflicts of interest

None disclosed.
